# *Hemiboea suiyangensis* (Gesneriaceae): a new species from Guizhou, China

**DOI:** 10.3897/phytokeys.99.25265

**Published:** 2018-05-30

**Authors:** Shuwan Li, Mengqi Han, Xiaojie Li, Zhenyu Li, Xiaoguo Xiang

**Affiliations:** 1 Guangxi Institute of Botany, Guangxi Zhuangzu Autonomous Region and the Chinese Academy of Sciences, 541006, Guilin, China; 2 College of Biology & Pharmacy, Yulin Normal University, 537000, Yulin, China; 3 Emeishan Biological Resources Research Station, Sichuan Provincial Academy of Natural Resources Sciences, 614200, Emeishan, China; 4 State Key Laboratory of Systematic and Evolutionary Botany, Institute of Botany, Chinese Academy of Sciences, 100093, Beijing, China

**Keywords:** *Hemiboea*, limestone, morphology, taxonomy

## Abstract

The limestone areas in south China are a major biodiversity hotspot for terrestrial biomes. *Hemiboea*, with 34 species and 5 varieties, mainly distributed in south China, is one of the characteristic plant groups in limestone areas. *Hemiboea suiyangensis*, a new species of Gesneriaceae from limestone areas in Guizhou, China, is described and illustrated. The new species is easily distinguished from other *Hemiboea* species by having an oblique-infundibular corolla with an abaxially gibbous swelling on the upper half of the tube and with a densely villose throat and lower lobes. *Hemiboea suiyangensis* is similar to *H. omeiensis* W. T. Wang in the shape of the leaf blade, but differs from the latter by the shape of the petiole, involucre, calyx and corolla and the colour of the corolla. The conservation status of this species is considered to be “Critically Endangered” (CR) according to IUCN Red List Criteria.

## Introduction

*Hemiboea* C. B. Clarke originally contained about 23 species and 5 varieties and occurred in central and southern China, northern Vietnam and Japan (Ryukyu Islands), with a distribution centre in China (Weber 2004; Li and Wang 2004). Later, seven new species and one new variety were found in Guangxi (Li 2004; Li and Liu 2004; Xu et al. 2010, 2012; Wen et al. 2011, 2013; Pan et al. 2012; Zhou et al. 2013) and one new species is known to be present in Yunnan (Zhang et al. 2014). Based on molecular evidence and extensive morphological examinations, Huang et al. (2017) treated Hemiboea subcapitata var. pterocaulis Z.Y. Li as a distinct species. The genus *Metabriggsia* including two species was revised and merged within *Hemiboea* (Weber et al. 2011). In total, this genus comprises at least 34 species and 5 varieties.

During the field work on investigation of limestone cave plants in 2015, we collected a rare plant of *Hemiboea* from the limestone area in Guizhou. After consulting the Flora of China (Wang et al. 1998), the monograph of Gesneriaceae in China (Li and Wang 2004) and other existing literature on Gesneriaceae and examining herbarium specimens, we recognised that the species belongs to *Hemiboea*, but it was readily distinguished from all known species of *Hemiboea* based on morphological characters and represented a new species, which is described and illustrated here.

## Material and methods

All available specimens of *Hemiboea* stored in the herbaria (IBK, KUN and PE) in China were examined. The photographs were taken in the field. Morphological observations and measurements of the new species were carried out based on living plants, dry specimens and preserved materials. All morphological characters were studied with dissecting microscopes and are described using the terminology presented by Wang et al. (1998).

## Taxonomic treatment

### Hemiboea suiyangensis

Taxon classificationPlantaeLamialesGesneriaceae

Z.Y.Li, S.W.Li & X.G.Xiang sp. nov.

urn:lsid:ipni.org:names:60476482-2

Figs 1 , 2

#### Diagnosis.

*Hemiboea suiyangensis* is easily distinguished from other *Hemiboea* species by having an oblique-infundibular corolla with abaxial gibbous, swollen on the upper half of the tube, throat and lower lobes densely villose. The species is similar to *H. omeiensis* W.T.Wang (1982: 127) in the shape of the leaf blade, but can be distinguished by oblate involucre (vs. globose), unequal calyx segments (vs. equal) and corolla with densely villose throat and lower lobes, lemon-yellow outside (vs. corolla with glabrous throat and lower lobes, white outside) (Fig. 3, Table 1).

#### Type.

China. Guizhou province: Suiyang County, Xiangshuwan, growing in cave entrance of limestone hills, about 885 m, 10 Aug. 2015, *M. Q. Han and S. W. Li HMQ 881* (holotype: IBK!, isotypes: IBK!, PE!).

#### Perennial herbs.

Stems ascending, subterete, 20–45 cm tall, 3–5 mm in diam., simple, sparsely purple-spotted, glabrous, slightly juicy when fresh, nodes 4–7, not swollen. Leaves opposite, unequal to sub-equal in a pair, herbaceous; leave blade oblong-lanceolate, ovate-lanceolate or elliptic, 4.5–19 cm long, 2.2–8 cm wide, apex acuminate, rarely acute, margin repand-crenate, base usually oblique, one side narrowly cuneate to cuneate, the other side cuneate to rounded, adaxial surface green, sparsely pubescent, abaxial surface pale green to purple, glabrous; lateral veins 6–13 on each side of midrib, vermiform sclereids surrounding the vascular bundles, veinlets inconspicuous; petiole 1–8.5 cm long, about 2 mm in diam., almost terete, adaxially valleculate, margins erect and rounded, glabrous, purple or purple maculate. Cymes pseudoterminal, sometimes axillary, 3–9(–12)-flowered; peduncle 1–2 cm long, about 2 mm in diam., glabrous, purple-spotted; involucre oblate, apiculate, 2.3–3.3 cm in diam., pale green, glabrous; pedicle 3–5 mm long, about 2 mm in diam., glabrous. Calyx white, glabrous, 5-parted to base; segments linear or linear-lanceolate, 11–13 mm long, 2–3 mm wide, unequal, adaxial three longer, ca. 13 mm long, abaxial two shorter, ca. 10 mm long. Corolla oblique-infundibular, 3.2–4 cm long, outside lemon-yellow, densely glandular-pubescent, inside glabrous, purple-spotted at throat, densely glandular-pubescent inside adaxial gibbous side of the tube, densely villose at the throat and lower lobes, a pilose ring 4–6 mm above corolla base; tube 2.5–3 cm long, 2.5–4 mm in diam. above base, swollen on abaxial side of the upper half of tube, 12–14 mm in diam. at mouth, gibbous; limb two-lipped, adaxial lip 3–3.5 mm long, 2-lobed at apex, lobes equal, nearly semi-orbicular, abaxial lip 7–10 mm long, 3-parted, lobes subequal, oblong, expanded to reflexed after anthesis. Protandrous; stamens 2, abaxial, adnate to 16–17 mm above corolla base, included, glabrous; filaments narrowly linear, 11–13 mm long, about 1 mm wide; anthers ovate-elliptic, 3–3.8 mm long, about 2 mm wide, coherent at apex; staminodes 3, adaxial, adnate to 8–11 mm above corolla base, glabrous, unequal, lateral two narrowly linear, 9–11 mm long, about 1 mm wide, apex capitellate, separate, central one linear, 2–3 mm long, about 1 mm wide, apex truncate. Disc ring-like, lemon-yellow, 1.4–1.7 mm high, margin repand. Pistil 18.7–19 mm long; ovary linear, 8.7–9 mm long, glabrous, about 3 mm wide; style ca. 10 mm long, about 1 mm in diam., sparsely glandular-puberulent; stigma oblate.

#### Distribution.

Only known from the Xiangshuwan, Suiyang County, Guizhou province, China.

#### Phenology.

The new species was observed flowering from June to August.

#### Habitat and ecology.

*Hemiboea suiyangensis* grows on moist stone at the limestone cave entrances, next to the stream. The main companion species are: *Pilea notata* C. H. Wright, *Elatostema prunifolium* W.T. Wang, *Acorus gramineus* Solander ex Aiton, *Ficus tikoua* Bureau and *Impatiens chlorosepala* Hand.-Mazz.

**Figure 1.**
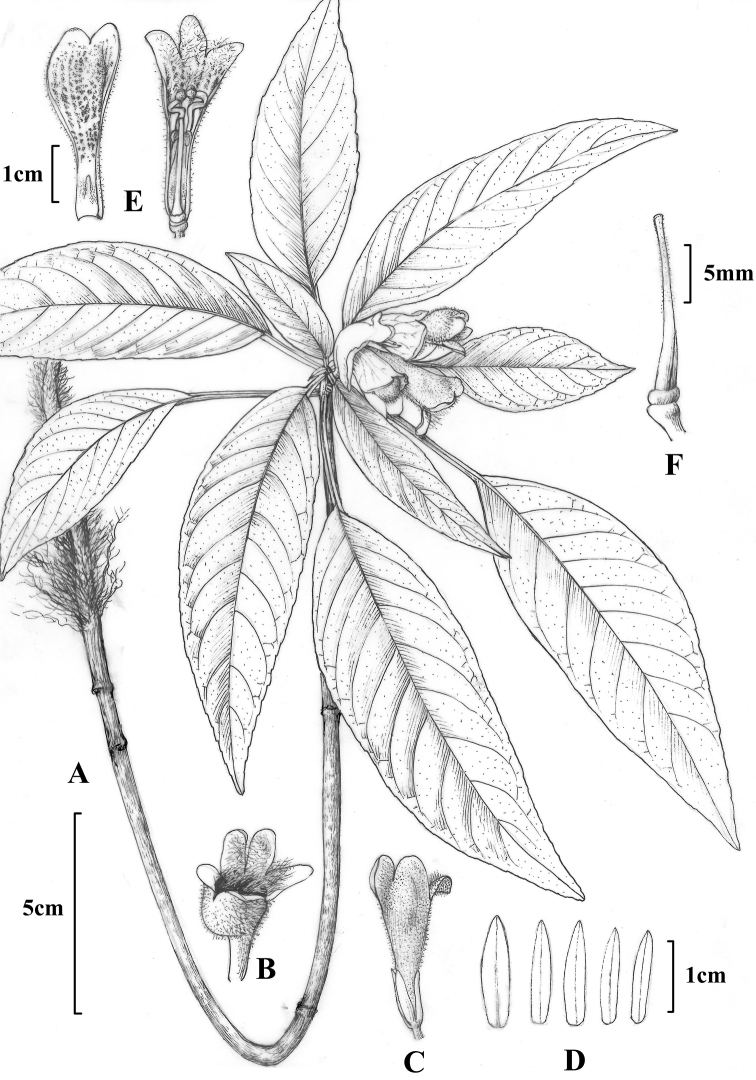
*Hemiboea suiyangensis*. **A** Plant **B, C** Flower outside view **D** Calyx segments **E** Flower inside view **F** Pistil.

**Figure 2.**
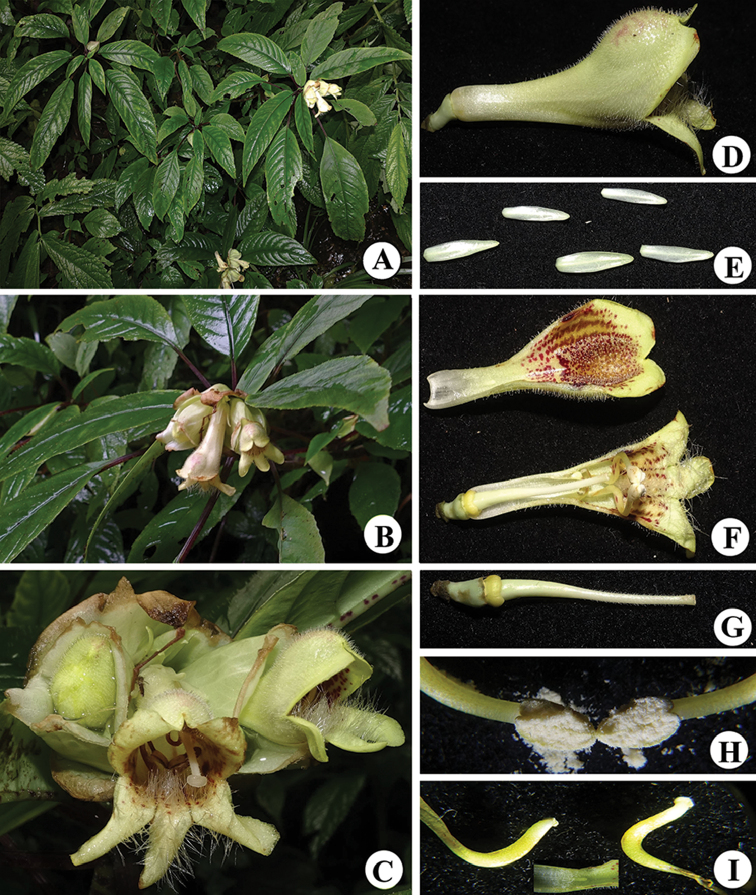
*Hemiboea suiyangensis*. **A** habits **B** flowering branch **C** flower face view **D** corolla side view **E** calyx **F** corolla inside view **G** pistillum **H** anthers **I** staminodia.

**Figure 3.**
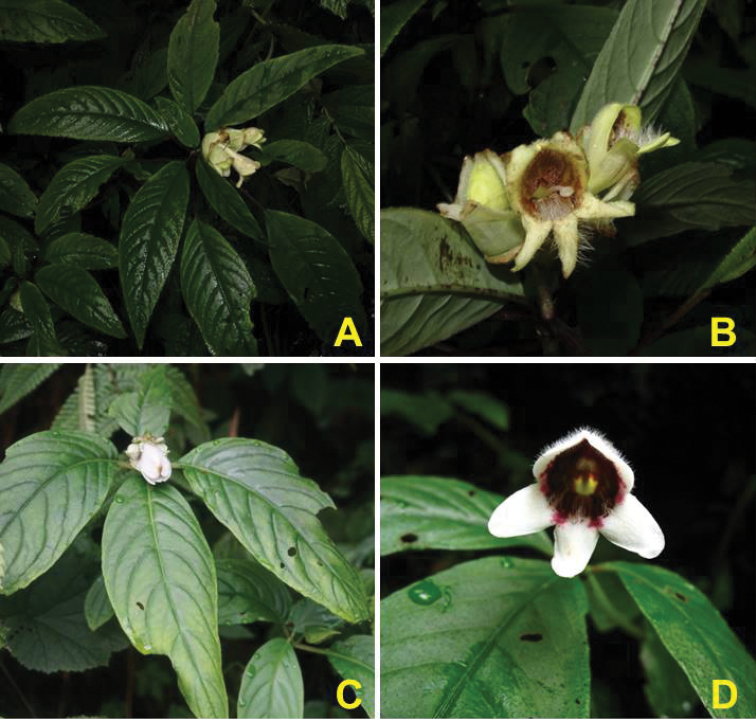
Comparisons between *Hemiboea suiyangensis* and *H. omeiensis*, **A–B** *Hemiboea suiyangensis*. Photographed by Meng-Qi Han **C–D** *H. omeiensis*. Photographed by Xiao-Jie Li.

**Table 1.** Morphological characters of *Hemiboea suiyangensis* and *H. omeiensis*.

**Characters**	***H. suiyangensis***	***H. omeiensis***
Petiole	1–8.5 cm long, almost terete, adaxial side valleculate, margins erect and rounded	0.3–3.5 cm long, almost (semi-) terete, adaxial side shallowly sulcate, margins spreading and compressed
Involucre	oblate, 2.3–3.3 cm in diam.	globose, 1.5–2.5 cm in diam.
Calyx segments	adaxial three, ca. 1.3 cm long; abaxial two, ca. 1 cm long	adaxial three, ca. 1.8 cm long; abaxial two, ca. 1.5 cm long
Corolla	oblique-infundibular, adaxially intumescent abruptly from half of tube, adaxial lip 3–3.5 mm long, abaxial lip 7–10 mm long, outside lemon-yellow densely villose at throat and lower lobes	tubular-infundibular, adaxially intumescent steadily from a quarter of tube, adaxial lip 8–10 mm long, abaxial lip 10–13 mm long, outside white, throat and lower lobes glabrous
Staminodes	3, lateral two 9–11 mm long, central one 2–3 mm long	3, lateral two 5–7 mm long, central one ca. 4 mm long
Disc	ring-like, lemon-yellow, 1.4–1.7 mm high	ring-like, white, 1–1.2 mm high

#### Etymology.

The species is named after the type locality, Suiyang County, Guizhou, China.

##### Key to *Hemiboea suiyangensis* and its alliance

**Table:** 

1	Petioles flattened adaxially, usually winged and connate perfoliate, especially upper pairs; vermiform sclereids dispersed in leaf mesophyll	***H. subcapitata* Clarke**
–	Petioles subterete, wingless, free; vermiform sclereids surrounding vascular bundles of leaf.	**2**
2	Corolla lemon-yellow outside, oblique-infundibular, with densely villose throat and lower lobes; disc lemon-yellow, 1.4-1.7 mm high	***H. suiyangensis* Z.Y.Li, S.W.Li & X.G.Xiang**
–	Corolla white outside, tubular-infundibular, with glabrous throat and lower lobes; disc white, 1–1.2 mm high.	**3**
3	Leaf blade with 8–10 lateral veins each side; cymes 3-6-flowered; corolla tube with dark-purple spots inside; staminodes 3	***H. omeiensis* W.T.Wang**
–	Leaf blade with 4–6 lateral veins each side; cymes 1-3-flowered; corolla tube with pink purple spots inside; staminodes 2	***H. gracilis* Franch.**

##### Proposed IUCN Conservation Status

According to field observations, *Hemiboea suiyangensis* has one known population of less than 20 mature individuals. The species is endemic in karst areas and is attributed to the diversity of cave plants. The population and habitat are susceptible to human activities, e.g. collection or deforestation. The species is considered to be “Critically Endangered” (CR) according to the IUCN Red List criteria (IUCN 2012), based on Criterion D, Population size estimated to number fewer than 50 mature individuals.

## Supplementary Material

XML Treatment for Hemiboea suiyangensis
